# Real-World Clinical Outcomes of Lebrikizumab in Moderate-to-Severe Atopic Dermatitis: A Report of Two Cases From Mexico

**DOI:** 10.7759/cureus.114629

**Published:** 2026-08-16

**Authors:** Grecia Zavala Villafuerte, Ana G Perez-Romero, Ana B Crocker Sandoval

**Affiliations:** 1 Internal Medicine, Tecnológico de Monterrey, Guadalajara, MEX; 2 Dermatology, Hospital Regional Valentín Gómez Farías, Instituto de Seguridad y Servicios Sociales de los Trabajadores del Estado (ISSSTE), Zapopan, MEX; 3 Dermatology, Hospital San Javier, Guadalajara, MEX

**Keywords:** atopic dermatitis (ad), biological therapy, case report series, interleukin-13, lebrikizumab, pruritus, quality of life (qol), real-world evidence

## Abstract

Atopic dermatitis (AD) is a chronic inflammatory skin disease associated with intense pruritus, impaired quality of life, and significant treatment challenges in patients with moderate-to-severe disease. Lebrikizumab, a monoclonal antibody targeting interleukin-13, has demonstrated efficacy in clinical trials; however, real-world evidence remains limited.

We present a case series of two women with long-standing moderate-to-severe AD refractory to multiple previous therapies, including topical corticosteroids, systemic corticosteroids, topical calcineurin inhibitors, crisaborole, and, in one case, cyclosporine. Baseline disease severity was assessed using the Eczema Area and Severity Index (EASI), SCORing Atopic Dermatitis (SCORAD), Dermatology Life Quality Index (DLQI), peak-pruritus Numerical Rating Scale (NRS), and body surface area (BSA). Both patients received lebrikizumab according to the approved dosing regimen and were followed for six weeks.

Substantial clinical improvement was observed in both cases. In Case 1, EASI decreased from 12.4 to 3.2, SCORAD from 38 to 11, DLQI from 18 to 4, and peak-pruritus NRS from 8 to 2. In Case 2, EASI decreased from 9.8 to 1.8, SCORAD from 32 to 8, DLQI from 16 to 3, and peak-pruritus NRS from 8 to 2. Improvement was particularly notable in highly visible and functionally sensitive anatomical areas, including the face, hands, and flexural regions. One patient reported improvement in pre-existing chronic conjunctivitis, while the second experienced improved asthma control during follow-up. No treatment-related adverse events were observed.

This case series provides additional real-world evidence supporting lebrikizumab as an effective and well-tolerated treatment for moderate-to-severe AD, with rapid improvement in disease severity, pruritus, and quality of life.

## Introduction

Atopic dermatitis (AD) is a chronic, relapsing inflammatory skin disease characterized by intense pruritus, eczematous lesions, and substantial impairment in quality of life. It is one of the most common inflammatory dermatoses worldwide, affecting up to 20% of children and 2%-10% of adults. Beyond its cutaneous manifestations, AD is associated with considerable psychosocial burden, sleep disturbances, and atopic comorbidities such as asthma, allergic rhinitis, and food allergies [1]. The pathogenesis of AD is multifactorial, involving epidermal barrier dysfunction, immune dysregulation, microbial dysbiosis, genetic susceptibility, and environmental factors [1,2]. Among the cytokines involved in disease pathogenesis, interleukin (IL)-13 plays a central role by promoting epidermal barrier impairment, type 2 inflammation, and pruritus [1,3].

Management of moderate-to-severe AD remains challenging despite the availability of topical therapies, phototherapy, and conventional systemic immunosuppressive agents. Advances in the understanding of AD immunopathogenesis have led to the development of targeted biologic therapies. Lebrikizumab is a humanized monoclonal antibody that selectively binds IL-13, thereby reducing type 2 inflammation and improving skin barrier function. Its efficacy and safety have been demonstrated in phase III clinical trials, including ADvocate 1, ADvocate 2, and ADhere, which reported significant improvements in disease severity, pruritus, and quality-of-life outcomes [2].

Despite encouraging clinical trial results, real-world evidence regarding lebrikizumab remains limited. Although phase III clinical trials provide robust evidence of treatment efficacy and safety under controlled conditions, real-world evidence complements these findings by providing information on treatment effectiveness and outcomes in routine clinical practice, where patient populations and treatment patterns may be more heterogeneous. Additional studies are needed to further characterize its effectiveness and safety in routine clinical practice. Therefore, we present a case series describing the clinical outcomes of two patients with moderate-to-severe AD treated at our institution with lebrikizumab.

## Case presentation

Case 1

**Figure 1:**
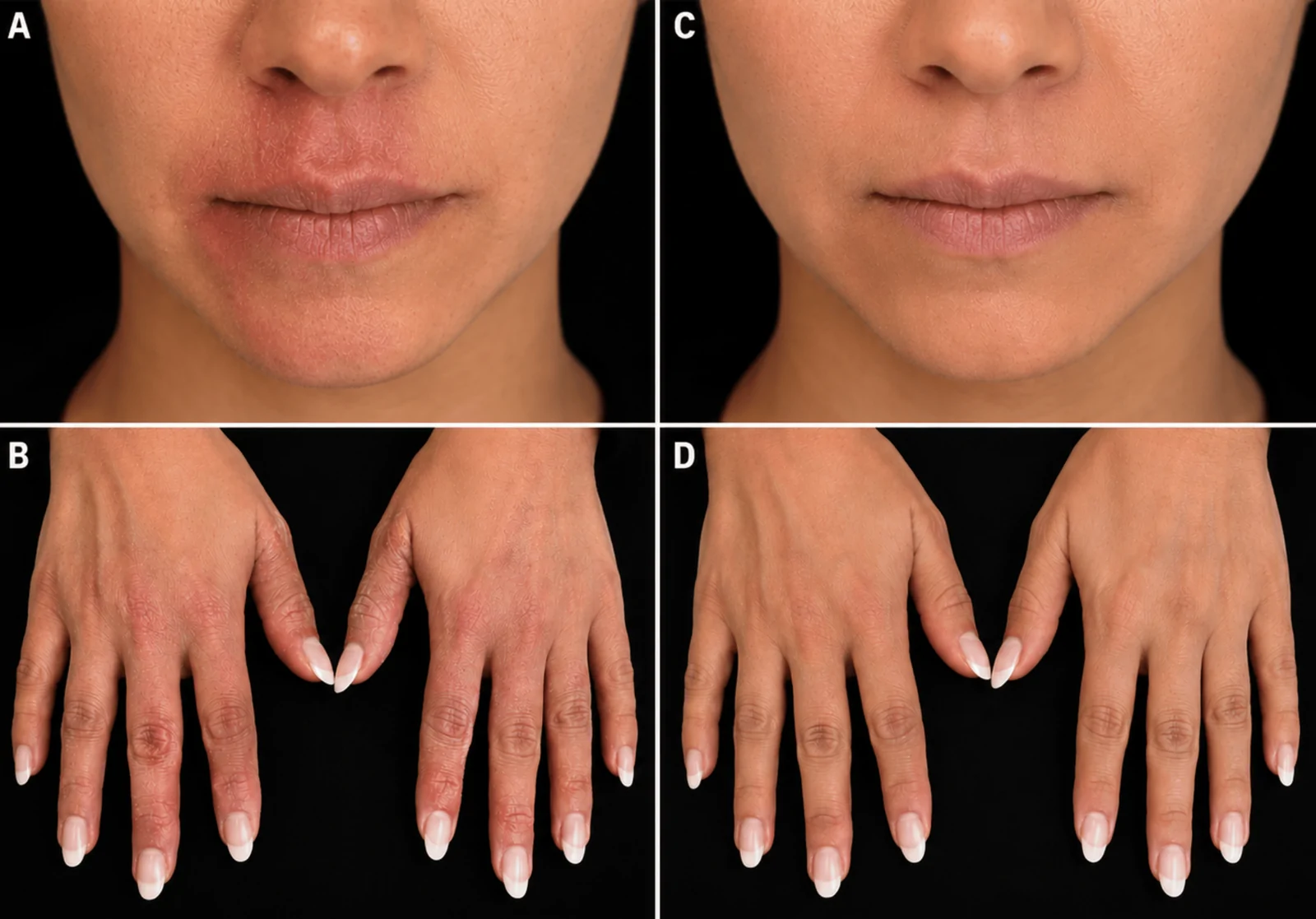
Case 1: Clinical improvement after six weeks of lebrikizumab. (A, B) Baseline perioral lesions and dorsal hand involvement. (C, D) Week 6 after initiation of lebrikizumab, showing marked improvement in perioral dermatitis and dorsal hand eczema.

A 36-year-old woman with a history of AD since the age of two years presented with persistent disease activity despite multiple previous therapies. Her medical history was notable for chronic conjunctivitis. The diagnosis of AD was established based on the chronic disease history and characteristic clinical findings. At baseline, the patient exhibited erythematous, scaly, and excoriated plaques involving the perioral region and dorsal aspects of the fingers and hands. Prior therapeutic interventions included topical corticosteroids, including clobetasol propionate and mometasone furoate; systemic corticosteroids (prednisone); topical calcineurin inhibitors (tacrolimus and pimecrolimus); and the phosphodiesterase-4 inhibitor crisaborole. Despite these treatments, the patient continued to experience persistent disease activity and inadequate symptom control. No formal washout period was performed before initiation of lebrikizumab. No concomitant systemic or topical treatment for AD was administered during the six-week follow-up period. Baseline disease severity was assessed using the Eczema Area and Severity Index (EASI) [4], Dermatology Life Quality Index (DLQI) [5], peak-pruritus Numerical Rating Scale (NRS) [6], Scoring Atopic Dermatitis (SCORAD) [7], and body surface area (BSA) [8], yielding values of 12.4%, 18%, 8%, 38%, and 6%, respectively. Clinical outcomes were assessed using physical examination and the aforementioned validated clinical outcome measures. Treatment with lebrikizumab was initiated on April 20, 2026, with a 500-mg loading dose at baseline, followed by maintenance therapy with 250 mg every two weeks thereafter. Clinical improvement was observed as early as week 6 of treatment. Follow-up examination demonstrated marked reductions in erythema, scaling, crusting, and excoriation, with near-complete resolution of facial lesions and substantial improvement of hand dermatitis. At week 6, the EASI score decreased to 3.2, DLQI to 4, peak-pruritus NRS to 2, SCORAD to 11, and BSA involvement to 1%. Additionally, the patient reported a noticeable reduction in conjunctivitis symptoms during treatment. No treatment-related adverse events were observed throughout the reported follow-up period. The clinical response was associated with substantial improvement in daily functioning and quality of life (Figures 1A-1D).

Case 2

**Figure 2:**
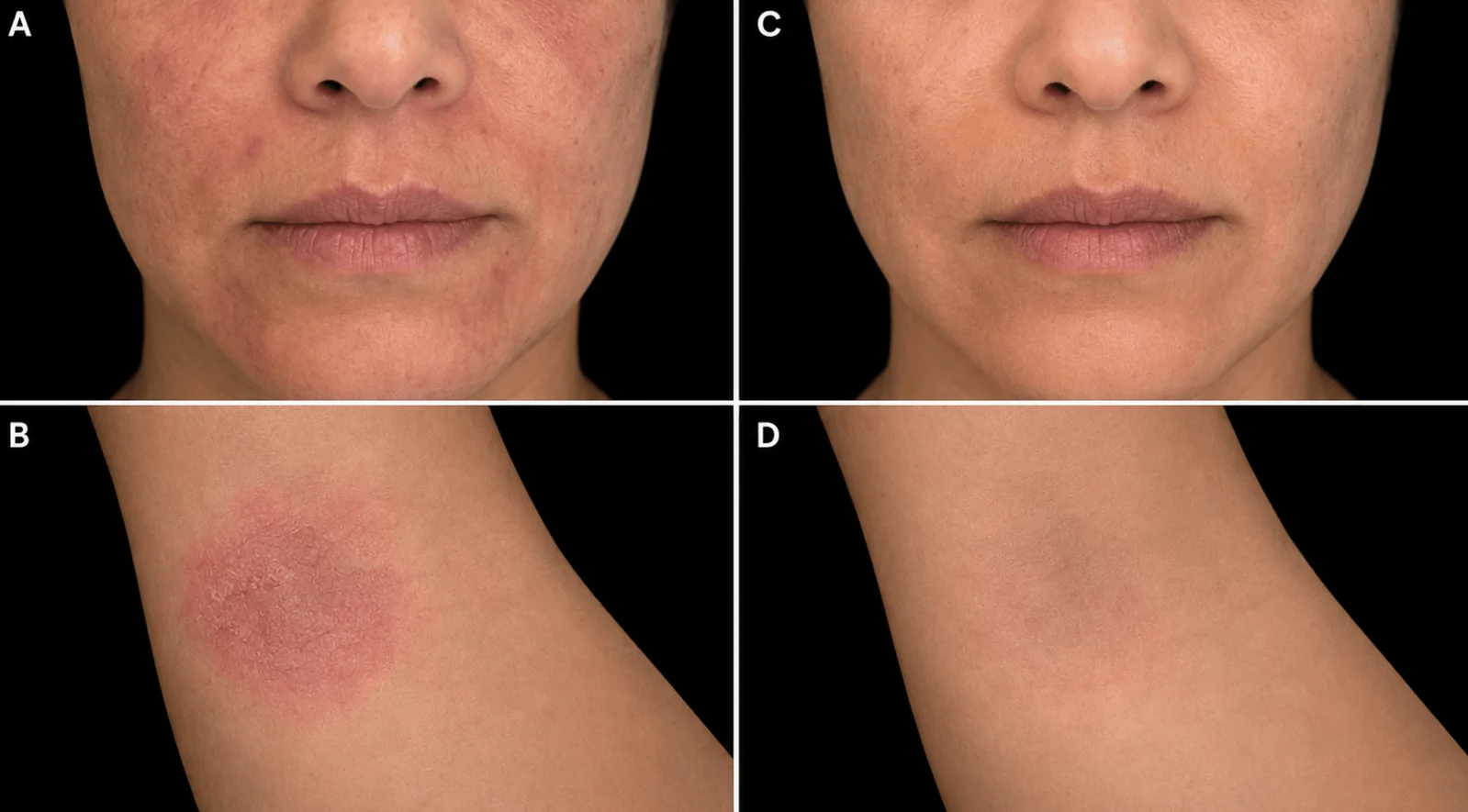
Case 2: Clinical improvement after six weeks of lebrikizumab. (A, B) Baseline facial lesions and antecubital fossa involvement. (C, D) Week 6 after initiation of lebrikizumab, showing marked improvement in facial dermatitis and resolution of the antecubital lesion.

**Figure 3:**
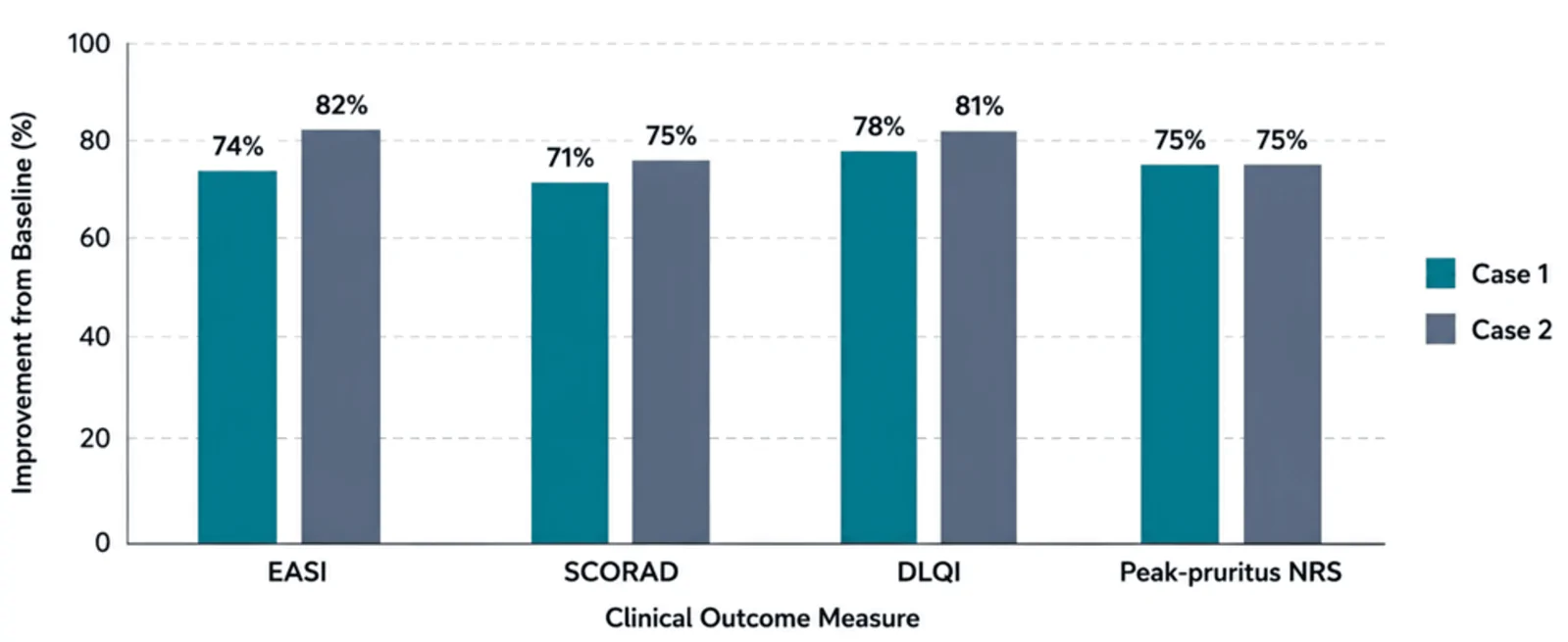
Percentage improvement in clinical outcome measures following six weeks of lebrikizumab treatment. Percentage improvement was calculated as ((baseline value − Week 6 value)/baseline value) × 100. BSA was not included because the week 6 BSA value for Case 2 was <1%, precluding precise calculation of percentage improvement. EASI, Eczema Area and Severity Index; SCORAD, Scoring Atopic Dermatitis; DLQI, Dermatology Life Quality Index; NRS, Numerical Rating Scale

A 44-year-old woman with a history of AD since the age of 14 years presented with persistent disease activity despite multiple previous therapies. Her medical history was notable for mild persistent asthma that was partially controlled at the time of lebrikizumab initiation, as well as food allergies. The diagnosis of AD was established based on the chronic disease history and characteristic clinical findings. At baseline, the patient exhibited active eczematous dermatitis involving flexural and facial areas. Clinical examination revealed erythematous, infiltrated plaques affecting the antecubital fossae, accompanied by facial involvement with periorbital erythema and chronic inflammatory changes. Previous treatments included clobetasol propionate 0.05% ointment, mometasone furoate 0.1% cream, the phosphodiesterase-4 inhibitor crisaborole, systemic corticosteroids, and cyclosporine at 3-5 mg/kg/day, all of which resulted in inadequate disease control or insufficient long-term remission. No formal washout period was performed before initiation of lebrikizumab. No concomitant systemic or topical treatment for AD was administered during the six-week follow-up period. Baseline disease severity was assessed using the EASI [4], DLQI [5], peak-pruritus NRS [6], SCORAD [7], and BSA [8], yielding values of 9.8%, 16%, 8%, 32%, and 4%, respectively. Clinical outcomes were assessed using physical examination and the aforementioned validated clinical outcome measures. Treatment with lebrikizumab was initiated on April 5, 2026, with a 500-mg loading dose at baseline, followed by maintenance therapy with 250 mg every two weeks thereafter. Clinical improvement became evident during follow-up and was clearly established by week 6, with marked reductions in erythema, inflammation, and lesion extent. At week 6, near-complete resolution of the antecubital lesion was observed, together with substantial improvement in facial involvement. Disease severity scores improved to an EASI score of 1.8, DLQI score of 3, peak-pruritus NRS score of 2, SCORAD of 8, and BSA involvement of less than 1%. In addition to the marked cutaneous response, the patient reported noticeable improvement in asthma control beginning during the second week of treatment. During follow-up, only a single episode of nocturnal asthma exacerbation was reported. No treatment-related adverse events were observed. The patient tolerated treatment well and experienced substantial clinical improvement (Figures 2A-2D, 3, Table 1).

**Table 1:** Changes in disease severity, pruritus, and quality of life after six weeks of lebrikizumab therapy. Baseline and week 6 disease severity outcomes in two patients treated with lebrikizumab. Percentage improvement was calculated as ((baseline value − Week 6 value)/baseline value) × 100. For Case 2, BSA improvement is reported as >75% because the week 6 BSA was <1%, precluding precise calculation of the percentage change. EASI, Eczema Area and Severity Index; SCORAD, Scoring Atopic Dermatitis; DLQI, Dermatology Life Quality Index; NRS, Numerical Rating Scale; BSA, body surface area

Outcome	Case 1 baseline	Week 6	Improvement (%)	Case 2 baseline	Week 6	Improvement (%)
EASI	12.4	3.2	74	9.8	1.8	82
SCORAD	38	11	71	32	8	75
BSA (%)	6	1	83	4	<1	>75
Peak-pruritus NRS	8	2	75	8	2	75
DLQI	18	4	78	16	3	81

Consent

Written informed consent was obtained from both patients for publication of their clinical information and accompanying images. The patients reviewed and approved the publication of anonymized photographs. All efforts were made to protect patient identity and confidentiality in accordance with ethical standards.

## Discussion

AD is a chronic inflammatory skin disease characterized by recurrent eczematous lesions, intense pruritus, epidermal barrier dysfunction, and substantial impairment in quality of life [3]. Despite advances in therapeutic options, many patients continue to experience inadequate disease control with topical therapies and conventional systemic agents [2]. In the present case series, both patients had long-standing AD refractory to multiple prior treatments. Following initiation of lebrikizumab, both patients experienced marked reductions in disease severity, pruritus, and body surface area involvement, accompanied by substantial improvements in quality-of-life measures. Particularly noteworthy was the clinical response observed in highly visible and functionally sensitive anatomical regions, including the face, hands, and flexural areas, where disease burden is frequently underestimated by conventional severity indices. These findings support the clinical utility of selective IL-13 inhibition in patients with moderate-to-severe AD affecting highly visible and functionally sensitive anatomical sites.

The clinical responses observed in this series are supported by the current understanding of AD pathophysiology. The disease results from a complex interaction between genetic susceptibility, epidermal barrier abnormalities, environmental exposures, microbial dysbiosis, and immune dysregulation [3,9]. Among the cytokines involved in type 2 inflammation, IL-13 has emerged as a key mediator of disease activity. IL-13 contributes to epidermal barrier dysfunction through downregulation of structural proteins such as filaggrin, loricrin, and involucrin, promotes eosinophilic inflammation, facilitates pruritic signaling pathways, and contributes to skin remodeling and chronic inflammation [2,3]. Furthermore, IL-13 has been implicated in increased transepidermal water loss and persistent xerosis, which may remain present even in clinically non-lesional skin [2]. Lebrikizumab is a high-affinity monoclonal antibody that selectively binds soluble IL-13. By preventing the formation of the signaling IL-4Rα/IL-13Rα1 signaling complex, it inhibits downstream pathways involved in type 2 inflammation, epidermal barrier dysfunction, pruritus, and tissue remodeling. Importantly, lebrikizumab preserves IL-13 binding to IL-13Rα2, a decoy receptor involved in endogenous cytokine regulation and cytokine clearance, thereby maintaining physiological regulatory mechanisms [2].

The rapid reduction in erythema, scaling, infiltration, and pruritus observed in our patients is consistent with this mechanism of action. Particularly noteworthy was the substantial reduction in itch scores observed within six weeks of treatment in both cases, as pruritus remains the hallmark symptom of AD and one of the strongest determinants of impaired sleep, emotional distress, and reduced quality of life [2,3]. The improvement in DLQI observed in parallel with reductions in EASI and NRS further suggests that targeting IL-13 may translate into benefits that extend beyond objective lesion clearance.

Our observations are consistent with the increasing amount of real-world evidence supporting the effectiveness of lebrikizumab. In a recently published case series from the Czech Republic, Jandová et al. described rapid clinical improvement in patients with moderate-to-severe AD treated in routine clinical practice [1]. Similarly, both patients in our series demonstrated substantial reductions in disease severity, pruritus, and quality-of-life impairment after six weeks of therapy. Notably, improvement was observed in highly visible anatomical areas such as the face and hands, which frequently contribute disproportionately to psychosocial burden despite limited body surface area involvement.

The second patient had concomitant asthma and food allergies in addition to AD, a phenotype that aligns with the broader spectrum of type 2 inflammatory disease. Furthermore, the favorable response observed despite previous exposure to cyclosporine suggests that lebrikizumab may represent an effective targeted alternative for patients who fail to achieve adequate disease control with conventional systemic therapies. In addition to cutaneous improvement, Case 2 reported better asthma control during follow-up, with fewer nocturnal symptoms and no requirement for rescue inhaled corticosteroids. Although respiratory outcomes were not formally assessed, this observation may be relevant given the recognized role of IL-13 in type 2 inflammatory diseases. Further studies are needed to better characterize the impact of selective IL-13 inhibition in patients with coexisting atopic comorbidities.

An additional noteworthy observation was the improvement of pre-existing chronic conjunctivitis symptoms reported by the patient in Case 1. Although this finding does not establish a causal relationship and should be interpreted cautiously, it may be clinically relevant given the growing interest in ocular outcomes among patients receiving biologic therapies for AD. Ocular surface disease, particularly conjunctivitis, has been recognized as one of the most common adverse events associated with dupilumab therapy in patients with AD, giving rise to the concept of Dupilumab-associated ocular surface disease (DAOSD) [10]. In contrast, clinical studies evaluating lebrikizumab have generally reported a favorable ocular safety profile, with relatively low rates of treatment-emergent conjunctivitis [2]. The improvement observed in our patient is therefore intriguing, particularly considering her long-standing history of chronic conjunctivitis before treatment initiation. While no conclusions can be drawn from a single case, this observation raises the possibility that selective IL-13 inhibition may have distinct ocular effects compared with dual IL-4/IL-13 blockade and highlights an area of interest for future research in patients with pre-existing ocular comorbidities. 

Several limitations should be acknowledged. This series includes only two patients from a single center and therefore cannot establish efficacy or safety beyond descriptive observation. Follow-up duration was limited, precluding assessment of long-term disease control, durability of response, and delayed adverse events. In addition, the absence of a comparator group prevents definitive attribution of all observed improvements to treatment alone. Nevertheless, the consistency of response across multiple outcome measures, including EASI, BSA, pruritus NRS, and DLQI, strengthens the clinical relevance of our observations. Furthermore, the marked improvement observed in anatomically sensitive areas highlights a benefit that may not be fully captured by severity indices alone. Although larger prospective studies are required to confirm these observations, our findings further support the role of IL-13 inhibition as a targeted therapeutic strategy capable of addressing both the inflammatory and barrier dysfunction components of AD.

## Conclusions

This case series describes short-term clinical improvements associated with lebrikizumab treatment in two patients with moderate-to-severe AD in a real-world setting. Both patients demonstrated improvement in disease severity, pruritus, and quality of life after six weeks of treatment, including marked clinical improvement in anatomically sensitive areas such as the face and hands. No treatment-related adverse events were observed during the reported follow-up period. However, the small sample size, uncontrolled design, and short follow-up period preclude conclusions regarding the long-term effectiveness or safety of lebrikizumab. These findings provide preliminary real-world observations that complement evidence from controlled clinical trials and support the need for further studies involving larger patient populations, longer follow-up, and controlled designs to better characterize the effectiveness and safety of lebrikizumab in routine clinical practice.
